# Congenital Thrombotic Thrombocytopenic Purpura: Atypical Presentation and New ADAMTS 13 Mutation in a Tunisian Child

**DOI:** 10.4084/MJHID.2013.041

**Published:** 2013-06-03

**Authors:** A. Borgi, M. Khemiri, A. Veyradier, K. Kazdaghli, S. Barsaoui

**Affiliations:** 1 Pediatric A Department-Children’s Hospital of Tunis, Faculty of Medicine University of Tunis Elmanar; 2 Laboratory of hematology. Antoine Beclere-Clamart Hospital Paris-France

## Abstract

**Background:**

Congenital deficiency of ADAMTS13 is characterized by systemic platelet clumping, hemolytic anemia and multiorgan failure. Although, more than 100 mutations have been reported, atypical clinical presentation may be involved in diagnostic difficulties.

**Case report:**

A 2 year old Tunisian child presented with chronic thrombopenic purpura which failed to respond to corticosteroids. Hemolytic anemia with schistocytes, occurred ten months later, with no previous history of diarrhea or any neurological abnormality. Renal function and coagulation screening tests were normal. The count of platelet improved after fresh frozen infusion (FFP). Extensive investigations revealed a severe deficiency of ADAMTS 13 activity (level< 5%). Gene sequencing identified mutation in exon 18 of ADAMTS 13 gene. Prophylactic regimen with regular infusions of FFP was associated to favorable outcome.

**Conclusion:**

Early ADAMTS 13 activity testing and gene sequencing associated to precocious plasmatherapy are recommended to reduce morbidity and mortality of congenital TTP.

## Introduction

Congenital Thrombotic thrombocytopenic purpura (TTP) is a rare life-threatening systemic illness potentially fatal in the absence of plasma therapy. It’s a severe form of thrombotic microangiopathy characterized by systemic platelet clumping, hemolytic anemia and multiorgan failure. TTP results from a defect in von Willebrond factor (vWF) cleaving protease, called ADAMTS 13.^1^ Inheritance of congenital TTP has been thought to be autosomal recessive. More than 100 mutations were reported at homozygous or heterozygous state in the ADAMTS 13 gene.^2^ Here, we report a case of congenital TTP with atypical clinical presentation; in which, we describe a new ADAMTS 13 mutation identified in a Tunisian child.

## Case Report

A 2 year-old boy was referred to our pediatric department for a 10-month’s history of thrombopenic purpura which failed to respond to conventional therapy with corticosteroids. The patient was born from healthy and no consanguineous parents. The medical history revealed an episode of anemia at the age of 1 month which required blood transfusion but no previous history of diarrhea. On admission, physical examination showed, fever at 38°C, skin purpura and no neurological impairment. The platelet count was 9×10^9^/l; hemoglobin level was at 6.8g/dl with marked reticulocytosis. Direct coombs test was negative and the lactate dehydrogenase rate was at 2314 IU/l. The blood film revealed numerous schistocytes and microspherocytes. Mild proteinuria and hematuria were detected but the renal function was preserved. Coagulation tests were normal. Regarding, the absence of previous diarrhea, and renal insufficiency, the diagnosis of atypical HUS was reported. Hemoglobin level has normalized (10.9g/dl) after one blood transfusion while platelet count remained low at 35 ×10^9^/l. TTP was then suspected; the patient was treated with 20ml/kg of fresh –frozen plasma, which promptly resulted in total recovery of thrombocytopenia (152×10^9^/l). He was discharged after improvement. Twenty days later, he relapsed a new hemolytic episode which improved with a new plasmatherapy. Further investigations revealed a severe deficiency of ADAMTS 13 activity (level< 5%), ADAMTS 13 antigen was undetectable (< 65ng/l; kit American Diagnostica, Stamford, USA). No inhibitors for ADMTS 13 were found. For genetic analysis, all 29 exons with flanking intron-exon baoundaries of the ADAMTS 13gene were amplified and sequenced. The propositus was found to be homozygous for a new mutation: c 2203 G>T-p.Glu735X (domain TSP1-2) in exon 18. The patient’s family sequencing gene couldn’t be performed but no members had a history of a TTP-like disorder, thrombosis or bleeding episodes. Since, the diagnosis of congenital TTP was confirmed; the patient was placed on prophylactic regimen with regular infusions of FFP. During 4 years of follow-up, only one hemolytic crisis occurred at starting therapy. This event led us to reduce the intervals between infusions from 4 weeks to 3 weeks. Small volumes (10ml/kg) of FFP were able to prevent crises. Actually, at 6 years of age, the patient has a normal physical and mental development. The renal function is still normal.

## Discussion

We report here by the clinical and biological findings of TTP in a young Tunisian child which was misdiagnosed for 10 months. Our patient was symptomatic probably within one month of age. Clinical signs often develop in the patients during the newborn period or early infancy.^3^ In fact, the earliest and most frequently encountered clinical manifestation is severe hyperbilirubinemia with negative Coombs test soon after birth, which requires exchange blood transfusions.^4^ Our patient showed an atypical clinical course. Thrombocytopenia occurred at 14 months of age, leading to suspect ITP. Absence of hemolytic anemia may lead to misdiagnosis, such ITP or Evans syndrome.^5^,^6^ Hemolytic anemia with schistocytes associated to profound thrombocytopenia, were found at 2 years of age. The most common thrombotic microangiopathy in child is typical post diarrheal HUS. If renal involvement is present, the condition can easily be misdiagnosed as HUS. Our patient had normal renal function with no previous history of diarrhea. TTP was suspected with delay regarding the rarity of the disease, and the absence of fluctuating neurological signs or renal failure. The classic criteria for distinguishing between TTP and HUS are the predominance of neurological symptoms in TTP and renal failure in HUS.^7^ Neurological involvement can be observed in patients with diagnosis of HUS and a large proportion of patients classified as having TTP show renal failure.^7^ Because TTP occurs less frequently in children, pediatricians are not well informed about the spectrum of clinical symptoms and altered laboratory values, increasing the risk of misdiagnosis and possible fatal outcome.

Assays for measurement of ADAMTS-13 were developed in the late 1990s, and significant improvements have occurred in the testing protocols to allow them to be performed in routine hemostasis laboratories.^8^ In our case, ADAMTS 13 activity testing was performed at 25 months of age after the successful therapeutic test of FFP infusion. To our knowledge, only two. Tunisian children had been reported in the literature.^9^,^10^ The first child was a 25 months old male, presented with relapses of hemolytic anemia, thrombocytopenia and renal failure. He also had severe deficiency of ADAMTS 13 activity in absence of inhibitors.^9^ ADAMTS 13 mutation was not specified in this case. The second Tunisian case of congenital TTP was a 17 years old of age. His clinical course was also atypical; the diagnosis was made after 3 years of relapses hemolytic crisis. He had a new homozygous mutation in the catalytic domain of ADAMTS 13 and a good outcome with plasma infusions.^10^

## Conclusion

Congenital Thrombotic thrombocytopenic purpura (TTP) is a rare life-threatening condition in childhood. Early detection of ADAMTS13 deficiency and precocious plasmatherapy are crucial to reduce morbidity and mortality. Looking for the diagnosis should be indicated even in uncommon or incomplete clinical presentation as in absence of multiorgan failure.
